# Ranolazine inhibits Na_V_1.5-mediated breast cancer cell invasiveness and lung colonization

**DOI:** 10.1186/1476-4598-13-264

**Published:** 2014-12-11

**Authors:** Virginie Driffort, Ludovic Gillet, Emeline Bon, Séverine Marionneau-Lambot, Thibauld Oullier, Virginie Joulin, Christine Collin, Jean-Christophe Pagès, Marie-Lise Jourdan, Stéphan Chevalier, Philippe Bougnoux, Jean-Yves Le Guennec, Pierre Besson, Sébastien Roger

**Affiliations:** Inserm UMR1069, Nutrition, Croissance et Cancer, Université François-Rabelais de Tours, 10 Boulevard Tonnellé, Tours, 37032 France; Cancéropôle du Grand Ouest, Nantes, France; Inserm U1009, Institut Gustave Roussy, Villejuif, France; CHRU de Tours, Tours, France; Inserm, U966, Université François-Rabelais de Tours, Tours, France; UFR Sciences Pharmaceutiques, Université François-Rabelais de Tours, Tours, France; Inserm U1046, Université de Montpellier-1, Université de Montpellier-2, Montpellier, France; UFR Sciences et Techniques, Département de Physiologie Animale, Université François-Rabelais de Tours, Tours, France

**Keywords:** Na_V_1.5 voltage-gated sodium channels, Cancer cell invasiveness, Ranolazine, Metastases

## Abstract

**Background:**

Na_V_1.5 voltage-gated sodium channels are abnormally expressed in breast tumours and their expression level is associated with metastatic occurrence and patients’ death. In breast cancer cells, Na_V_1.5 activity promotes the proteolytic degradation of the extracellular matrix and enhances cell invasiveness.

**Findings:**

In this study, we showed that the extinction of Na_V_1.5 expression in human breast cancer cells almost completely abrogated lung colonisation in immunodepressed mice (NMRI nude). Furthermore, we demonstrated that ranolazine (50 μM) inhibited Na_V_1.5 currents in breast cancer cells and reduced Na_V_1.5-related cancer cell invasiveness *in vitro. In vivo*, the injection of ranolazine (50 mg/kg/day) significantly reduced lung colonisation by Na_V_1.5-expressing human breast cancer cells.

**Conclusions:**

Taken together, our results demonstrate the importance of Na_V_1.5 in the metastatic colonisation of organs by breast cancer cells and indicate that small molecules interfering with Na_V_ activity, such as ranolazine, may represent powerful pharmacological tools to inhibit metastatic development and improve cancer treatments.

**Electronic supplementary material:**

The online version of this article (doi:10.1186/1476-4598-13-264) contains supplementary material, which is available to authorized users.

## Findings

Breast cancer is the primary cause of death by cancer in women worldwide and patients mostly die because of metastases appearance and development [1] which rely in part on the ability of cancer cells to degrade and migrate through extracellular matrices (ECM). Currently, there is no treatment for specifically inhibiting metastases development. Voltage-gated sodium channels (Na_V_) are essential for action potential firing and as such are characteristic of excitable cells [2]. However, different Na_V_ isoforms have been found in non-excitable epithelial human cancer biopsies and cells, such as in breast [3, 4], lung [5–7], prostate [8], cervix [9], ovarian [10, 11] and colon cancer [12], and their function, through persistent currents at the membrane potential, enhances degradation of ECM [5, 13–15]. Notably, the Na_V_1.5 isoform is abnormally expressed in breast cancer biopsies, while it is not in non-cancerous mammary tissues [14], and its level of expression is associated with lymph node invasion, the development of metastases and a reduced survival of patients [3, 16, 17]. In cancer cells, it is expressed as a neonatal splice variant showing a 7-amino acid substitution in the segments S3 and S4 of the domain I (DI-S3-S4) of the protein compared to the adult variant that shows a particular pharmacology [18] and was proposed to serve as a metastatic marker [16]. Na_V_1.5 is functional at the plasma membrane of highly invasive breast cancer cells [3, 16, 17], and its activity maintains a pro-invasive phenotype [15], related to “mesenchymal migration” [19]. Indeed, while the complete mechanism involved is not yet elucidated, Na_V_1.5 activity was shown *i)* to control Src kinase activity, cortactin phosphorylation (Y421) and the subsequent polymerisation of actin filaments, *ii)* to increase the activity of the Na^+^/H^+^ exchanger type 1 (NHE-1), thus enhancing the efflux of protons and the proteolytic activity of extracellularly-released acidic cysteine cathepsins B and S [13, 20]. Altogether, these results indicated that Na_V_1.5 promotes the invadopodial activity of breast cancer cells and the invasion of the surrounding ECM [15]. Molecules reducing its activity, such as tetrodotoxin, reduce cancer cell invasiveness *in vitro*[3, 14, 18]. Correlatively, molecules that increase its activity, such as veratridine, enhance ECM invasion [13]. However, to the best of our knowledge, the importance of Na_V_1.5 expression, and the relevance for its pharmacological inhibition, on the metastatic organ colonisation by breast cancer cells have never been reported so far. Ranolazine is an antiarrhythmic drug indicated for the treatment of chronic angina that was approved by the Food and Drug Administration (FDA) in 2006. While it is proposed to have several pharmacological actions, its best characterized one is the selective inhibition of late sodium currents [21]. This leads to a steeper Na^+^ gradient which, by increasing the activity of the Na^+^/Ca^2+^ exchanger (NCX), reduces calcium overload, and improves ventricular relaxation in pathological conditions associated with cardiac ischemia [22].

In this study we investigated how Na_V_1.5 expression in human breast cancer cells affected metastatic colonisation of organs in immunodepressed mice, and whether its pharmacological inhibition by ranolazine reduced cancer cell invasiveness both *in vitro* and *in vivo*.

**Figure 1:**
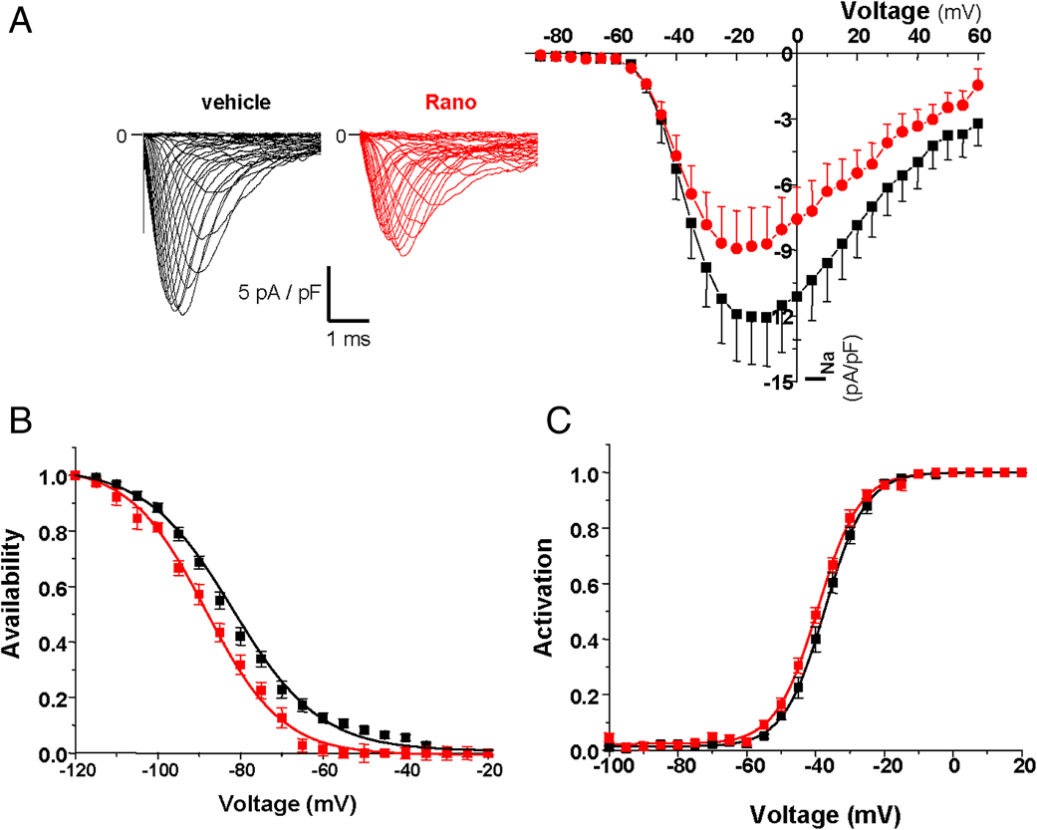
**Ranolazine inhibits sodium current in human breast cancer cells.** Sodium currents (INa) from MDA-MB-231 breast cancer cells stably expressing null target shRNA (shCTL) were studied in voltage-clamp mode with the whole-cell configuration of the patch clamp technique. **A**, Left, representative INa-voltage traces obtained from one cell before (vehicle) and after 50 μM ranolazine treatment (Rano). Right, mean ± s.e.m. steady-state INa-voltage relationships obtained from cancer cells before and after incubation with 50 μM ranolazine (n = 12 cells) from a holding potential of −100 mV. There is as statistical difference between the two conditions for voltages ranging from −35 to +40 mV (p < 0.001, Wilcoxon test). **B**, Availability-voltage relationships obtained in presence (red trace) or not (vehicle, black trace) of 50 μM ranolazine. There is a significant leftward shift of the availability-voltage relationship in presence of ranolazine (p < 0.001). The half (1/2)-inactivation voltage was shifted from −84.1 ± 1.4 mV to −90.3 ± 1.7 mV in absence and presence of ranolazine, respectively. **C**, Activation-voltage relationships obtained in presence (red trace) or not (vehicle, black trace) of 50 μM ranolazine. There is a significant leftward shift of the activation-voltage relationship in presence of ranolazine, and the 1/2-activation voltage was shifted from −37.1 ± 1.0 mV to −39.2 ± 0.6 mV in absence and presence of ranolazine, respectively. (p < 0.01, Wilcoxon test).

**Figure 2:**
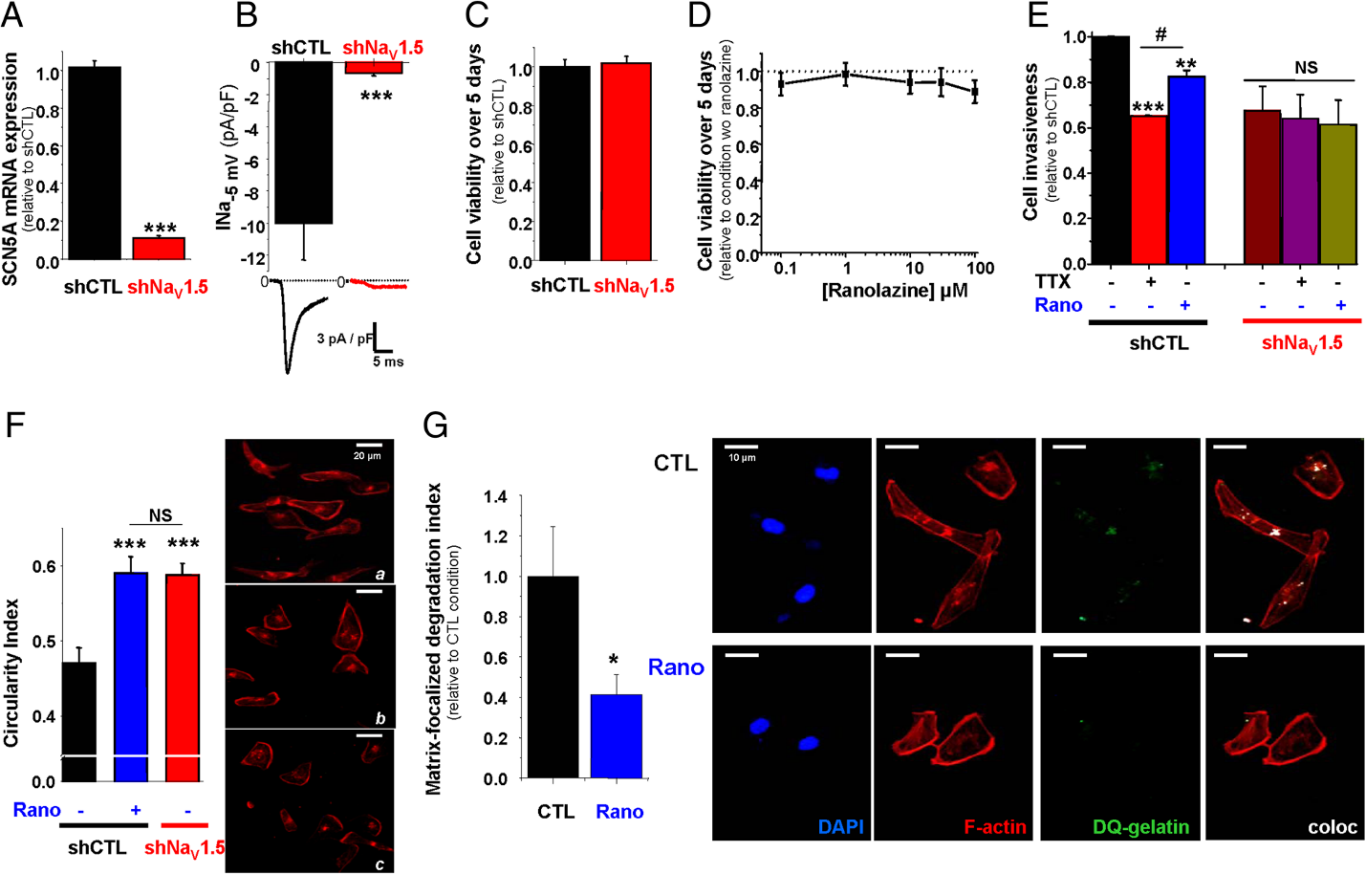
**Ranolazine inhibits the Na**_**V**_ **1.5-mediated breast cancer cell invasiveness** ***in vitro.*** **A**, *SCN5A* mRNA expression assessed by real-time qPCR in shCTL and shNa_V_1.5 cells (n = 10 separate experiments) and compared with a Mann–Whitney test. **B**, Mean ± s.e.m. peak INa recorded in 23 shCTL cells and in 20 shNa_V_1.5 cells under a depolarization from −100 to −5 mV (Mann–Whitney test). Representative currents are shown underneath. **C**, shCTL and shNa_V_1.5 cell growth and viability after 5 days, expressed relative to the shCTL cell line (n = 3 independent experiments). **D**, Cell viability of shCTL after 5 days of growth in presence of increasing concentrations of ranolazine, from 0.1 to 100 μM, and expressed relative to the control condition without ranolazine (vehicle). **E**, Effect of 30 μM tetrodotoxin (TTX) or 50 μM ranolazine (Rano) on shCTL and shNa_V_1.5 human breast cancer cell invasiveness (Kruskal-wallis analysis followed by a Dunn’s test). **F**, shCTL and shNa_V_1.5 cells were cultured for 24 h on a Matrigel™-composed matrix treated with 50 μM ranolazine (Rano) or not. F-actin cytoskeleton was stained with phalloidin-AlexaFluor594. A cell circularity index was calculated using ImageJ© software (n = 138–238 cells analysed, Mann–Whitney test). **G**, shCTL cells were cultured on a Matrigel™-composed matrix containing DQ-Gelatin® for 24 h in presence or not of 50 μM ranolazine. A “Matrix-Focalized-degradation activity index” was calculated as being the number of pixels corresponding to the co-localization of F-actin condensation areas (F-actin cytoskeleton was stained with phalloidin-Alexa594) and focal spots of DQ-gelatin proteolysis (coloc) (7). Results are expressed relative to the control condition (CTL, N = 534 cells) without ranolazine (Rano, N = 375 cells) and compared using Mann–Whitney test. Representative pictures are shown on the left. Statistical significance is indicated as: *p <0.05; **p < 0.01 and ***p < 0.001. NS stands for not statistically different.

Highly invasive MDA-MB-231 human breast cancer cells express mRNA for Na_V_1.5, Na_V_1.6 and Na_V_1.7 channels [16], but only Na_V_1.5 channels are functional at the plasma membrane and are giving rise to transient inward sodium currents (INa) under voltage-clamp procedures [13] (see Additional file 1: Material and methods). INa-voltage (INa-V) protocols were performed using the whole-cell configuration of the patch clamp technique from MDA-MB-231-Luc cells modified to stably express a null-target small hairpin RNA (shCTL). The INa-V relationship, obtained from a holding potential of −100 mV, indicated a threshold of activation around −60 mV and maximal current of −12.1 ± 2.2 pA/pF at a voltage of −10 mV (Figure 1A). The acute application of ranolazine (50 μM) significantly reduced the maximal amplitude to −8.7 ± 1.7 pA/pF (p < 0.001). This decrease in the maximal current amplitude was associated with a significant leftward shift of the availability-voltage relationship (Figure 1B). The half (1/2)-inactivation voltage was shifted from −84.1 ± 1.4 mV to −90.3 ± 1.7 mV (p < 0.001) in absence and presence of ranolazine, respectively. The activation-voltage relationship was significantly modified (Figure 1C), and the 1/2-activation voltage was shifted from −37.1 ± 1.0 mV to −39.2 ± 0.6 mV (p < 0.01). Therefore, ranolazine reduced efficiently the activity of the neonatal Na_V_1.5 isoform expressed in human breast cancer cells. This isoform is the only one to be functional in breast cancer cells [13, 16] and we selected a population of cells stably expressing a small hairpin RNA targeting its expression (shNa_V_1.5) after lentiviral transduction. This led to a significant 89 ± 1% decrease of Na_V_1.5 mRNA expression (Figure 2A), resulting in the complete disappearance of sodium currents in almost all cancer cells (Figure 2B), with no effect on cell viability (Figure 2C). Before assessing the effect of ranolazine in reducing cancer cell invasiveness, we addressed a possible cytotoxic effect of its application. Figure 2D indicates that ranolazine, incubated for 5 days in a range of concentration from 0.1 to 100 μM had no effect on cell viability. It was then used at 50 μM in the 24 h invasion experiment with Matrigel™-coated filters (Figure 2E). In shCTL cells, cell invasiveness was reduced by 35 ± 4% with the total inhibition of Na_V_1.5 currents with 30 μM tetrodotoxin (TTX), and by 18 ± 3% with ranolazine. In comparison to shCTL cells, shNa_V_1.5 cancer cells, which do not express Na_V_1.5, had a reduced invasiveness of 33 ± 10%. In shNa_V_1.5 cells, both TTX and ranolazine were ineffective to further reduce cell invasiveness, suggesting that ranolazine was specific in inhibiting Na_V_1.5-related invasion. Na_V_1.5 expression and activity was recently shown to control the acquisition of a pro-invasive phenotype, by maintaining a spindle-shape morphology and by controlling the ECM proteolysis by MDA-MB-231 cancer cells [15]. We found that ranolazine increased the circularity of shCTL cells, thus decreasing the pro-invasive morphology, to the same extent as the complete extinction of Na_V_1.5 expression (Figure 2F). Furthermore, ranolazine reduced the focal ECM degradative activity of shCTL cells by 58.6 ± 10.0% (Figure 2G). This activity, which is related to the invadopodial activity, was monitored as being the release of fluorescence from DQ-gelatin at focal sites of F-actin polymerisation as previously described [15].

**Figure 3:**
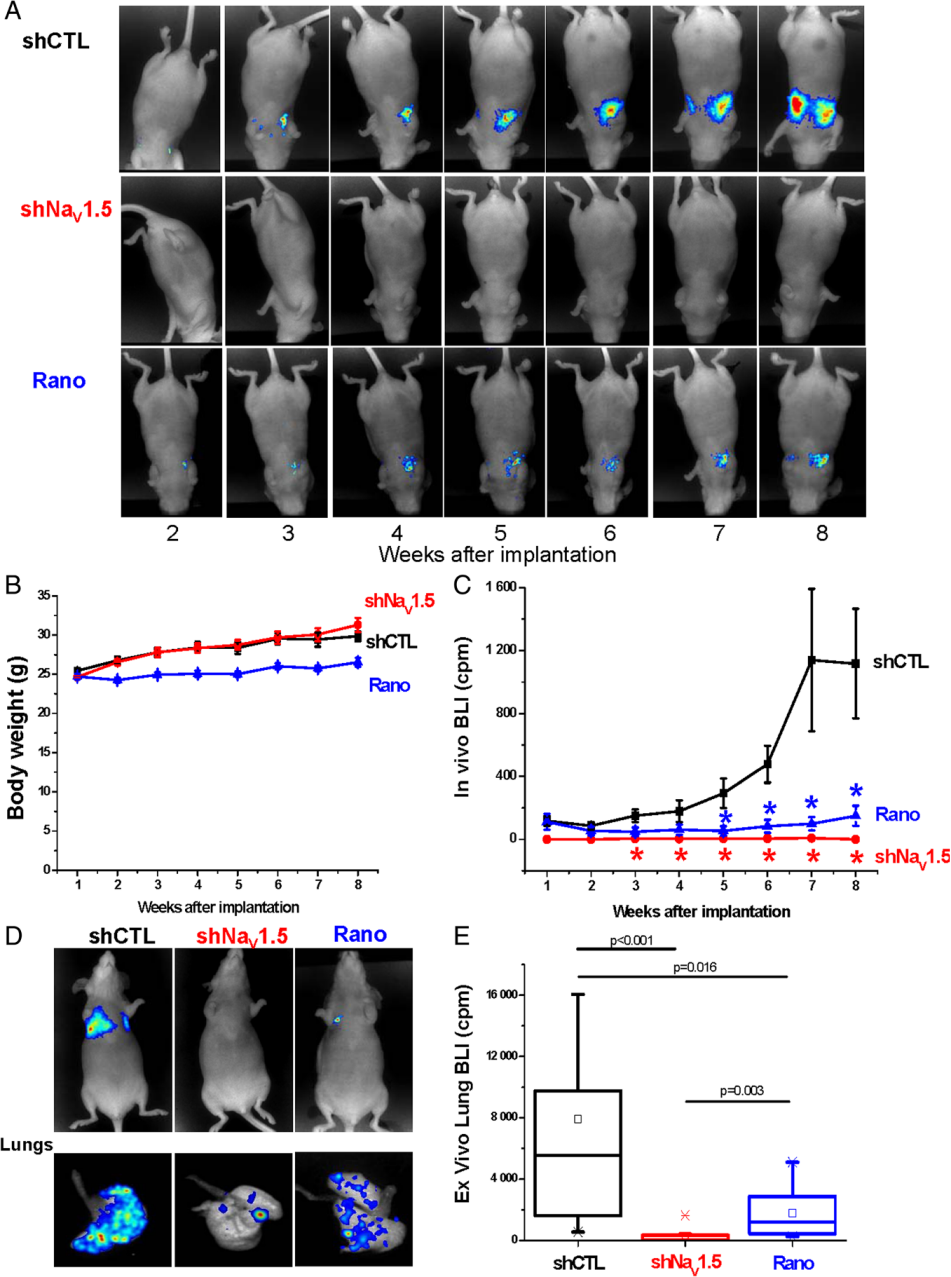
**Na**_**V**_ **1.5 suppression, or ranolazine treatment, inhibit metastatic lung colonisation by breast cancer cells. A**, Representative bioluminescent imaging (BLI) measurement performed in the same NMRI nude mouse per condition from week 2 to week 8 after cancer cell injection. Mice were injected with shCTL MDA-MB-231-Luc cells (shCTL), or with shNa_V_1.5 MDA-MB-231-Luc cells (shNa_V_1.5) or with shCTL MDA-MB-231-Luc cells and treated (5 days/week) with ranolazine (50 mg/kg) (Rano) or vehicle (shCTL, shNa_V_1.5). **B**, Evolution of mice body weight during the experiments in the same conditions than in A. **C**, Mean *in vivo* BLI value (expressed in cpm) as a function of time recorded in the whole body of mice coming from the three groups indicated previously (shCTL, n = 18; shNa_V_1.5, n = 12; Rano, n = 8) (Statistical significance is indicated as: *p <0.05, Kruskal-Wallis analysis followed by Dunn’s test). **D**, Representative BLI at completion of the study (8^th^ week after cells injection), in whole animals and *ex vivo* after lung isolation. **E**, BLI quantification of excised lungs. Box plots indicate the first quartile, the median, and the third quartile, squares indicate the mean (shCTL, n = 18; shNa_V_1.5, n = 12; Rano, n = 8) (Kruskal-Wallis analysis followed by Dunn’s test).

Because Na_V_1.5 was proposed to promote metastases development from breast tumours, we assessed the importance of its expression in human breast cancer cells for the colonisation of organs. ShCTL or shNa_V_1.5 cells, both expressing the luciferase gene, were injected in the tail vein of NMRI nude mice. A third experimental group was set with mice injected with shCTL cells and receiving a daily intraperitoneal injection of ranolazine (50 mg/Kg – 5 days per week). The colonisation of mice organs by human cancer cells was followed *in vivo*, every week for a total duration of 8 weeks, by bioluminescent imaging (BLI) after luciferin injection (Figure 3A). There was no statistical difference in the evolution of animal body weights between the three groups (Figure 3B). BLI performed in living animals indicated that shCTL cells, which express Na_V_1.5, strongly colonised and developed into the chest area (which was the case for 17 out of 18 mice). In contrast, shNa_V_1.5 cells led to a very weak signal (1/12 mice) or no signal at all (11/12 mice) in the chest area. Ranolazine, which inhibited Na_V_1.5 currents (Figure 1), significantly reduced total BLI signal in mice injected with shCTL cells. *In vitro*, ranolazine treatment did not interfere with luciferase activity in shCTL cells (Additional file 2: Figure S1) indicating that the BLI signal recorded was indeed strongly correlated with the abundance of cancer cells in mice organs. In this experimental group, BLI signal was recorded in 5 out of 8 mice (Figure 3C). At completion of the study, mice were sacrificed and isolated organs (lungs, brain, liver, bones from rachis/ribs and legs) were analysed *ex vivo*. In the shCTL group, all mice showed lung colonisation (18/18) and a small proportion also had bioluminescent signal in rachis and ribs (2/18) and in leg bones (2/18). In the shNa_V_1.5 group, 7 mice out of 12 had lung colonisation and one (1/12) had bioluminescent signal in rachis and ribs. In the ranolazine group, although 8/8 mice presented lung colonisation, bioluminescence was dramatically reduced by 77 ± 8%, at a level similar to the experimental suppression of Na_V_1.5 (inhibition of lung BLI by 97 ± 2%) (Figure 3D, 3E). In the ranolazine group, mice did not show BLI signal in other organs.

While it is now well-established that Na_V_ channels are anomalously expressed in several epithelial tumours and are associated with metastasis occurrence and patient mortality [12, 16, 17, 23], the consequence of their expression on metastatic organ colonisation was not demonstrated so far. To our knowledge, this study is the first to clearly establish a link between Na_V_1.5 expression in human breast cancer cells and the colonisation of lungs *in vivo*. Furthermore, this study using ranolazine, a drug that is clinically used, shows that the pharmacological inhibition of Na_V_ channels could be effective in reducing metastastic colonisation with no apparent toxic effect. In conclusion, this study opens a new therapeutical concept for the management of cancer disease. Inhibitors of Na_V_ channels, already approved for other clinical use such as antiarrhythmics, anticonvulsants [17] or anaesthetics [24], or new molecules that are even more effective in blocking neonatal variants, could be of high interest in the prevention and/or reduction of metastatic spreading of cancer cells at the diagnosis of the primary tumour.

## Electronic supplementary material

Additional file 1: **Materials and methods.** (DOC 45 KB)

Additional file 2: Figure S1: MDA-MB-231-shCTL cells expressing luciferase gene were seeded at different densities then treated for 24h with Ranolazine (50 μM, red circles) or not (black squares). (PDF 11 KB)

## Abbreviations

BLI: Bioluminescent imaging
ECM: Extracellular matrix
INa: Sodium currents
Na_V_: Voltage-gated sodium channels
TTX: Tetrodotoxin.
