# Supplementary material for: Ranolazine inhibits NaV1.5-mediated breast cancer cell invasiveness and lung colonization
Source: Mol Cancer. 2014 Dec 11;13:264. doi: 10.1186/1476-4598-13-264 (PMC4295566; doi:10.1186/1476-4598-13-264)
Supplement: Supplementary file 1 — Additional file 1: Materials and methods. (DOC 45 KB) [file 12943_2014_1462_MOESM1_ESM.doc]

**Materials and methods**

***Inhibitors, chemicals and antibodies -***Tetrodotoxin was purchased from Latoxan (France). Fluorescent probes were purchased from Tebu-Bio (France). Ranolazine ([(+)N-(2,6-dimethylphenyl)-4(2-hydroxy-3-(2-methoxyphenoxy)-propyl)-1-piperazine acetamide dihydrochloride]), other drugs and chemicals were purchased from Sigma-Aldrich (France). Ranolazine was prepared in phosphate buffer saline (PBS).

***Cell culture and cell lines -***MDA-MB-231-Luc human breast cancer cells, stably expressing the luciferase gene, were cultured in DMEM supplemented with 5% FCS, and grown at 37°C in a humidified 5% CO_2_ incubator. We constructed a lentiviral vector encoding a short hairpin RNA (shRNA) specifically targeting human *SCN5A* transcripts using the same protocol as previously described ^25^. The sequence encoding shNa_V_1.5 was obtained by DNA polymerase fill-in of two partially complementary primers: 5'-GGATCCCCAAGGCACAAGTGCGTGCGCAATTCAAGAGA-3’ and 5’-AAGCTTAAAAAAAGGCACAAGTGCGTGCGCAATCTCTTGAA-3’. We also constructed a lentiviral vector expressing an untargeted shRNA (pLenti-shCTL), using the following primers: 5′-GGATCCCCGCCGACCAATTCACGGCCGTTCAAGAGACG-3′ and 5’-AAGCTTAAAAAGCCGACCAATTCACGGCCGTCTCTTGAACG-3’. These experiments resulted in shNa_V_1.5 and shCTL cell lines that had similar luciferase activity *in vitro*.

***RNA extraction, Reverse transcription, and real-time qPCR*** - Total RNA extraction from MDA-MB-231 cells was performed by using NucleoSpin® RNA Isolation (Macherey-Nagel, France). RNA yield and purity were determined by spectrophotometry and only samples with an A260/A280 ratio above 1.6 were kept for further experiments. Total RNA were reverse-transcribed with PrimeScript RT reagents® kits (Takara, Ozyme, France). Real time PCR experiments were performed as previously described ^13^. Primers had the following sequences (expected sizes), for Na_V_1.5: forward 5’-CACGCGTTCACTTTCCTTC-3’ and reverse 5’- CATCAGCCAGCTTCTTCACA-3’ (208 bp), and for HPRT1: forward 5’-TTGCTGACCTGCTGGATTAC-3’ and reverse 5’- TATGTCCCCTGTTGACTGGT-3’ (119 bp).

***Cellular electrophysiology* -** Patch pipettes were pulled from borosilicate glass to a resistance of 4-6 MΩ. Sodium currents were recorded, in whole-cell configuration, under voltage-clamp mode using an Axopatch 200B amplifier (Axon Instrument, USA) as already described ^13^. External Sodium Solution (in mM): NaCl 140, KCl 4, MgCl_2_ 1, CaCl_2_ 2, D-Glucose 11.1, HEPES 10, adjusted to pH 7.4. Intrapipette solution (in mM) : KCl 130, NaCl 15, CaCl_2_ 0.37, MgCl_2_ 1, Mg-ATP 1, EGTA 1, HEPES 10, adjusted to pH 7.2.

***Cell viability*** - Cells were seeded at 4x10^4^ cells per well in a 24-well plate and were grown for a total of 5 days. Culture media (containing or not ranolazine at the concentrations indicated in the figure legends) were changed every day. Viable cell number was measured by the tetrazolium salt assay as previously described and normalised to the control condition, without ranolazine or shCTL ^13^.

***In vitro Invasion Assays*** – Cell invasiveness was analysed as previously described ^20^ using culture inserts with 8-µm pore size filters covered with Matrigel™ (Becton Dickinson, France).

***Epifluorescence imaging*** – Cells were cultured for 24h on coverslips coated with a matrix composed of Matrigel™ (4 mg/mL) and containing or not DQ-gelatin® (25 µg/mL) as a fluorogenic substrate of gelatinases. They were then washed in PBS, fixed with 3.7% ice-cold paraformaldehyde in PBS. Cells were permeabilized with a solution containing 50 mM NH_4_Cl, 0.2% BSA, 0.02% saponin and saturated in 3% BSA and 3% normal goat serum. F-actin was stained with phalloidin-AlexaFluor594. Epifluorescence microscopy was performed with a Nikon TI-S microscope and analysed using both NIS-BR software (Nikon, France) and ImageJ© software 1.38I (<http://rsbweb.nih.gov/ij>). A circularity index was calculated as being 4π·Area/Perimeter^2^. A value approaching 0 indicates an increasingly elongated shape while a value of 1.0 indicates a perfect circle. The number of pixels corresponding to the co-localization of F-actin condensation areas and focal spots of DQ-gelatin proteolysis (excitation wavelength: 495 nm, emission wavelength: 515 nm) were quantified per cell, giving a matrix-focalized-degradation index.

***Experimental model for metastatic colonisation*** - NMRI Nude Mice (Charles River laboratories) were bred and housed at the In Vivo platform of the Cancéropôle Grand Ouest at Inserm U892 (Nantes) under the animal care license n° 44278. The project was approved by the national ethical committee (ref n°00085.01). Unanaesthetized 6-week-old female mice were placed into a plastic restraining device, and 2 × 10^6^ MDA-MB-231-Luc cells (shCTL/shNa_V_1.5) suspended in 150 μL PBS were injected into the lateral tail vein through a 25-gauge needle. Mice received 50 mg/kg ranolazine or vehicle (PBS) intraperitonealy, 5 days per week for 8 weeks. All mice were weekly assessed using whole-body bioluminescent imaging (Φimageur^TM^, BIOSPACE Lab). Mice were injected intraperitoneally 150 mg/kg D-luciferin potassium salt (Interchim) and anesthetized with isoflurane. BLI signals were collected 3-5 min after injection, in real time until saturation plateau was reached in the ventral and dorsal positions. Photons emitted by cancer cells were counted and expressed in counts per minute (cpm). At necropsy, *ex vivo* BLI measurement for each collected organ was performed within 15 min after D-luciferin injection.

***Statistical analyses -***Statistical analyses were performed using SigmaStat 3.0 (Systat software *inc*.). Statistical significance is indicated as: *, p <0.05; **, p<0.01 and ***, p<0.001. NS stands for not statistically different.

***Reference:***

25. Jelassi B, Chantome A, Alcaraz-Perez F, Baroja-Mazo A, Cayuela ML, Pelegrin P, Surprenant A, Roger S: **P2X(7) receptor activation enhances SK3 channels- and cystein cathepsin-dependent cancer cells invasiveness.** *Oncogene* **2011;30**:2108-22.
