# Supplementary material for: Ranolazine inhibits NaV1.5-mediated breast cancer cell invasiveness and lung colonization
Source: Mol Cancer. 2014 Dec 11;13:264. doi: 10.1186/1476-4598-13-264 (PMC4295566; doi:10.1186/1476-4598-13-264)
Supplement: Supplementary file 2 — Additional file 2: Figure S1: MDA-MB-231-shCTL cells expressing luciferase gene were seeded at different densities then treated for 24h with Ranolazine (50 μM, red circles) or not (black squares). (PDF 11 KB) [file 12943_2014_1462_MOESM2_ESM.pdf]

Supplementary Fig. 1

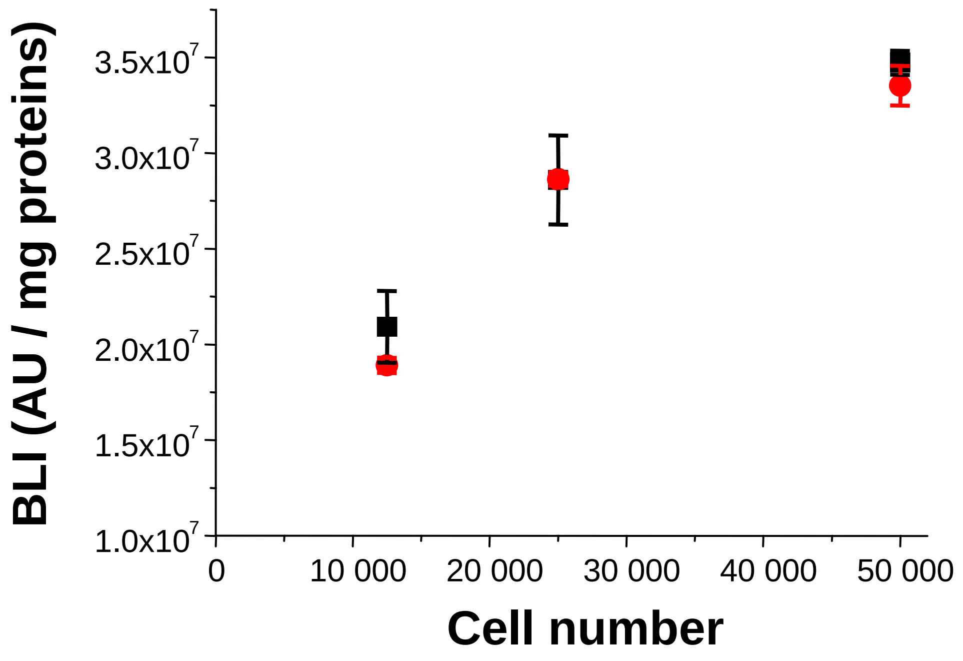

Supplementary Figure 1 :
MDA-MB-231-shCTL cells expressing luciferase gene were seeded at different densities then treated for 24h with Ranolazine (50 µM, red circles) or not (black squares). After 24h growth, cells were lysed and the bioluminescent signal (BLI) was measured after the addition of luciferin (Luciferase Assay System kit, Promega) and expressed as arbitrary units relativized to the total amount of proteins (AU/mg proteins).Results are expressed as mean ± sem. There was no difference between the two conditions.
